# Plasma amyloid-β42/40 and apolipoprotein E for amyloid PET pre-screening in secondary prevention trials of Alzheimer’s disease

**DOI:** 10.1093/braincomms/fcad015

**Published:** 2023-01-24

**Authors:** Nicholas C Cullen, Shorena Janelidze, Erik Stomrud, Randall J Bateman, Sebastian Palmqvist, Oskar Hansson, Niklas Mattsson-Carlgren

**Affiliations:** Clinical Memory Research Unit, Department of Clinical Sciences Malmö, Faculty of Medicine, Lund University, 202 13 Lund, Sweden; Clinical Memory Research Unit, Department of Clinical Sciences Malmö, Faculty of Medicine, Lund University, 202 13 Lund, Sweden; Clinical Memory Research Unit, Department of Clinical Sciences Malmö, Faculty of Medicine, Lund University, 202 13 Lund, Sweden; Memory Clinic, Skåne University Hospital, 205 02 Malmö, Sweden; Department of Neurology, Washington University School of Medicine, St. Louis, MO 63110, USA; Clinical Memory Research Unit, Department of Clinical Sciences Malmö, Faculty of Medicine, Lund University, 202 13 Lund, Sweden; Memory Clinic, Skåne University Hospital, 205 02 Malmö, Sweden; Clinical Memory Research Unit, Department of Clinical Sciences Malmö, Faculty of Medicine, Lund University, 202 13 Lund, Sweden; Memory Clinic, Skåne University Hospital, 205 02 Malmö, Sweden; Clinical Memory Research Unit, Department of Clinical Sciences Malmö, Faculty of Medicine, Lund University, 202 13 Lund, Sweden; Department of Neurology, Skåne University Hospital, 221 85 Lund, Sweden; Wallenberg Center for Molecular Medicine, Lund University, 221 84 Lund, Sweden

**Keywords:** Alzheimer’s disease, clinical trials, plasma biomarkers, amyloid, PET

## Abstract

The extent to which newly developed blood-based biomarkers could reduce screening costs in secondary prevention trials of Alzheimer’s disease is mostly unexplored. We collected plasma amyloid-β42/40, apolipoprotein E ε4 status and amyloid PET at baseline in 181 cognitively unimpaired participants [the age of 72.9 (5.3) years; 61.9% female; education of 11.9 (3.4) years] from the Swedish BioFINDER-1 study. We tested whether a model predicting amyloid PET status from plasma amyloid-β42/40, apolipoprotein E status and age (combined) reduced cost of recruiting amyloid PET + cognitively unimpaired participants into a theoretical trial. We found that the percentage of cognitively unimpaired participants with an amyloid PET + scan rose from 29% in an unscreened population to 64% [(49, 79); *P* < 0.0001] when using the biomarker model to screen for high risk for amyloid PET + status. In simulations, plasma screening also resulted in a 54% reduction of the total number of amyloid PET scans required and reduced total recruitment costs by 43% [(31, 56), *P* < 0.001] compared to no pre-screening when assuming a 16× PET-to-plasma cost ratio. Total savings remained significant when the PET-to-plasma cost ratio was assumed to be 8× or 4×. This suggests that a simple plasma biomarker model could lower recruitment costs in Alzheimer’s trials requiring amyloid PET positivity for inclusion.

## Teaser

Cullen *et al.* demonstrated that combining plasma amyloid-β42/40 with *APOE* status and age could be used to effectively screen cognitively unimpaired individuals in the Swedish BioFINDER study for amyloid PET abnormality. These results add to the evidence that this panel could greatly improve recruitment for clinical trials.

## Graphical Abstract

**Graphical abstract:**
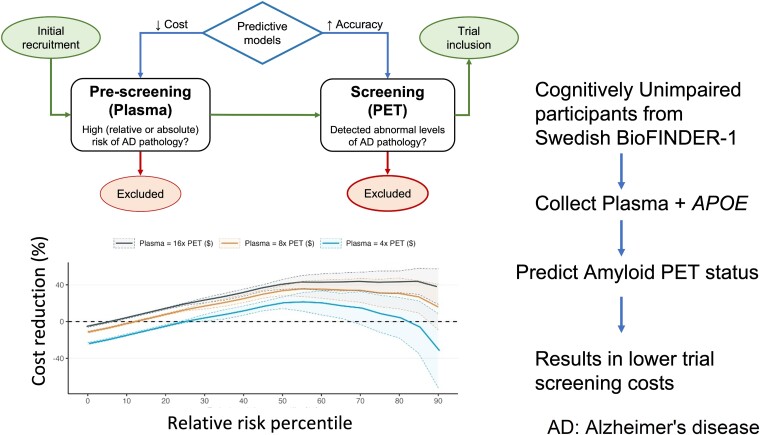

## Introduction

Alzheimer’s disease is the most common form of dementia and is expected to increase in prevalence during the coming decades due to a growing elderly population.^1^ A disease-modifying compound that lowers brain amyloid β (Aβ) load in mild Alzheimer’s disease recently gained regulatory approval,^2^ and other candidate treatments also look promising.^3^ However, clinical trials have been largely unsuccessful when targeting patients at later stages of Alzheimer’s disease. These failures have led to a shifting focus towards patients at earlier disease stages where cognitive symptoms are minor and Alzheimer’s disease pathology may be present but not widespread.^4,5^

In the future, the target population of Alzheimer’s disease therapies is likely to include elderly individuals who are at risk for Alzheimer’s disease but do not exhibit any cognitive impairment.^6^ Such secondary prevention trials in cognitively unimpaired (CU) individuals may still require confirmation of Aβ pathology before the start of treatment to ensure target engagement, although recent trials in the symptomatic phase of the disease suggest that tau pathology could also be useful for selecting an appropriate trial population.^3^ The gold standard of detecting Aβ pathology is through PET scanning or cerebrospinal fluid collection, but these modalities are likely to remain prohibitively expensive as a front-line screening tool for secondary prevention (both in trials and in clinical practice) since the prevalence of Aβ pathology is estimated to be around 25% in enriched Alzheimer’s disease study cohorts and as low as 15% in the general population (depending on age).^7^

Therefore, there is a need for pre-screening tools which can effectively and affordably identify CU individuals at high risk for Aβ pathology before a more invasive and expensive measurement is obtained for confirmation. Effective pre-screening biomarkers could lower overall recruitment costs by reducing the number of negative (i.e. normal) Aβ PET scans which result in exclusion from trials. Recently established plasma biomarkers of Alzheimer’s disease show great promise in filling this role as inexpensive predictors of abnormal Aβ pathology as measured by PET.^5^ In particular, plasma Aβ42/Aβ40—considered to directly reflect Aβ pathology—has been shown to provide significant prognostic value for Alzheimer’s disease-related outcomes in a CU population.^8^ Apolipoprotein E (*APOE)* is also the strongest genetic risk predictor for the risk of Alzheimer’s disease in healthy elderly individuals and may be included in prognostic models which are utilized for early screening of the disease.^9,10^

**Figure 1:**
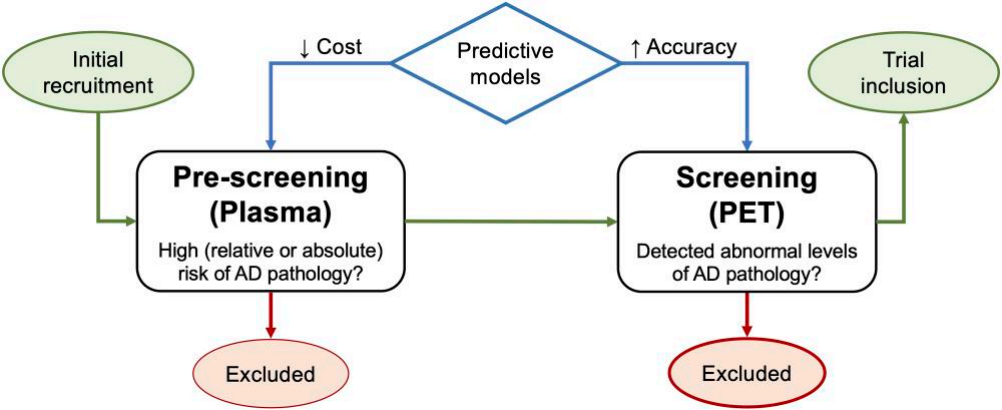
**A schematic workflow of clinical trial recruitment in secondary prevention trials.** This figure shows a schematic workflow of clinical trial recruitment in secondary prevention trials of Alzheimer’s disease. It demonstrates how both pre-screening and screening phases of trial recruitment can benefit from clinical prediction models. It also shows how the priority of pre-screening is to have a low cost model, while the priority of screening is to have high accuracy biomarkers which can truly detect individuals who have abnormal Alzheimer’s disease pathology.

Although it is clear that these biomarkers are individually related to Aβ PET risk in a CU population, more studies are needed to fully understand how a panel of non-invasive biomarkers can be realistically operationalized for trial screening while still acknowledging the eventual necessity of Aβ PET collection. One recent paper demonstrated the efficiency of combining plasma Aβ42/Aβ40, *APOE* status and age to predict Aβ PET status in a mixed group of individuals with and without cognitive impairment.^11^ In this study, we expand on this approach to predict the risk of abnormal Aβ PET scans using this same plasma biomarker panel but focusing on a CU population and exploring a novel cost-benefit perspective throughout a variety of clinical trial designs. Validation of Aβ screening panels in CU populations is of high importance to facilitate its establishment in clinical care and secondary prevention trials. We hypothesized that this biomarker panel could reduce the cost of recruiting Aβ PET + CU participants in a simulated clinical trial. We also placed our risk prediction model into the context of a larger framework of trial recruitment in Alzheimer’s disease involving separate ‘pre-screening’ and ‘screening’ phases (see Fig. 1).

## Materials and methods

### Study participants

Participants from the Swedish BioFINDER-1 (Biomarkers for Identifying Neurodegenerative Disorders Early and Reliably; clinical trial no. NCT01208675, www.biofinder.se) cohort were included in the present analysis. The CU group consisted of (i) cognitively normal (CN), healthy control participants with no objective evidence of cognitive impairment at baseline and (ii) participants with subjective cognitive decline (SCD) who were referred to the memory clinic for investigation but deemed to not have any cognitive impairment after undergoing an extensive neuropsychological battery.^12,13^ The inclusion criteria were the following: (i) age ≥60 years for CN, 60–80 years for SCD; (ii) absence of objective cognitive impairment as assessed by a physician with a special interest in cognitive disorders; (iii) mini-mental state examination score of at least 28 for CN participants and at least 24 for SCD participants at screening visit; (iv) fluent in Swedish and (v) does not fulfil the criteria for mild cognitive impairment or dementia according to DSM-5. Exclusion criteria included (i) a significant unstable systemic illness that makes it difficult to participate in the study and (ii) current significant alcohol or substance misuse.

The study was approved by the institutional review board at Lund University and written informed consent was received from all participants. All data were collected between July 2008 and June 2019. Only participants with complete data for all variables were included in the present study.

### Biomarker and genetic measurements

Plasma Aβ42/Aβ40 was measured at baseline at the Bateman laboratory at Washington University using a method which has been previously described.^14^ Briefly, plasma samples were spiked with 15N-Aβ40 and 15N-Aβ42 for use as analytical reference standards. Aβ42 and Aβ40 isoforms were immunoprecipitated using a monoclonal anti-Aβ mid-domain antibody (HJ5.1, anti-Aβ13-28). Liquid chromatography-tandem mass spectrometry (LC-MS/MS) and analysis of MS data were then performed.^14^ Average intra-assay coefficient of variation was 0.72% and the average inter-assay coefficient of variation was 3.46%. There were no failed samples.

To facilitate easier interpretation of results, plasma Aβ42/Aβ40 levels were negated (i.e. multiplied by −1) so that higher values indicate worsening Aβ pathology. Aβ pathology in the brain was measured at baseline using 18F-flutemetamol PET conducted on a Philips Gemini TF 16 scanner. A global neocortical composite standardized uptake ratio (SUVR) was calculated for each individual using cerebellar cortex as the reference region. A pathologically abnormal (‘positive’) Aβ PET scan was defined as SUVR > 0.742 as defined previously through mixture modelling.^15^ Further, *APOE* genotype was measured at baseline and, due to a low number of *APOE* ε4 homozygotes in the sample, was treated as a binary variable indicating presence of at least one ε4 allele or not.

### Statistical analysis

A logistic regression model was first fit with Aβ PET status [normal (‘PET-’) versus abnormal (‘PET+’)] as an outcome and with plasma Aβ42/Aβ40, *APOE* status and age as predictors. We tested whether using this fitted model to choose which CU participants should undergo amyloid PET scanning could reduce the cost to recruit 500 Aβ PET + CU participants into a theoretical clinical trial compared to obtaining Aβ PET scans on everyone. A value of 500 participants was selected to reflect reasonable sample sizes for recruitment in clinical trials in this population.^16^

We did this by comparing the percentage of Aβ PET + participants in the entire study population (representing a scenario without pre-screening) with the expected percentage of Aβ PET + participants found when using the biomarker model for pre-screening across a range of risk thresholds from 0 to 90%. This analysis was done first using a *relative risk* approach where an individual’s risk value was calculated relative to the entire study population, and also using an *absolute risk* approach where risk was represented as the absolute probability of being Aβ PET+ . In other words, relative risk is interpreted as an individual’s risk relative to others in the study population (e.g. 40% relative risk means the individual is in the 40th percentile in the population) while the absolute risk is interpreted as an individual’s risk relative in absolute terms (e.g. 40% absolute risk means the individual has a 40% probability of being Aβ PET positive). Subsequently, we calculated the total cost of recruitment at each risk threshold across a range of PET-to-plasma cost ratios (4×, 8×, 16×).

While the primary goal was to validate a pre-specified and well-established panel, a data-driven approach to variable selection was also undertaken where non-significant and non-trending variables were removed from the screening model.

All analysis was done in the R programming language (v4.0.3) and all statistical tests were two-sided with *P* < 0.05. All confidence intervals (CIs) and *P*-values were derived from 1000 trials of bootstrap sampling to extrapolate what model performance would be on new data. The performance of the logistic regression model was additionally validated using 5-fold cross-validation.

## Results

### Study participants

A total of 52 of 180 (28.9%) participants had abnormal Aβ PET scans at baseline and 80 of 180 (44.4%) participants had SCD. The distribution of Aβ PET positivity in the SCD group (32 of 80; 40%) compared to the CN group (20 of 100; 20%) was significantly different (*P* = 0.005). The average age was 73.0 ± 5.3 years, with 111 (61.7%) female participants and an average educational attainment of 11.9 ± 3.3 years. The cohort is further described in Table 1.

**Table 1:** Cohort characteristics

	Level	Value
*n*		180
Diagnosis [*n* (%)]	CN	100 (55.6)
	SCD	80 (44.4)
Age [mean (sd)]		73.01 (5.29)
Sex [*n* (%)]	Male	69 (38.3)
	Female	111 (61.7)
Education [mean (sd)]		11.90 (3.32)
*APOE* ε4 alleles [*n* (%)]	0	119 (66.1)
	1	51 (28.3)
	2	10 (5.5)
Aβ PET, SUVR [mean (sd)]		0.73 (0.15)
Aβ PET, status [*n* (%)]	Normal	128 (71.1)
	Abnormal	52 (28.9)
PACC, baseline [mean (sd)]		−0.13 (3.63)
Plasma Aβ42/Aβ40 [mean (sd)]		0.13 (0.01)
Plasma P-tau217 [mean (sd)]		0.17 (0.14)
Plasma NfL [mean (sd)]		21.33 (9.70)
Plasma GFAP [mean (sd)]		1247.75 (621.75)

This table describes the cohort used for analysis. All continuous values are reported as mean and standard deviation while all categorical values are reported as count and percentage.

CN, cognitively normal; GFAP, glial fibrillary acidic protein; PACC, preclinical Alzheimer cognitive composite; SCD, subjective cognitive decline.

### Prediction of amyloid PET status

**Figure 2:**
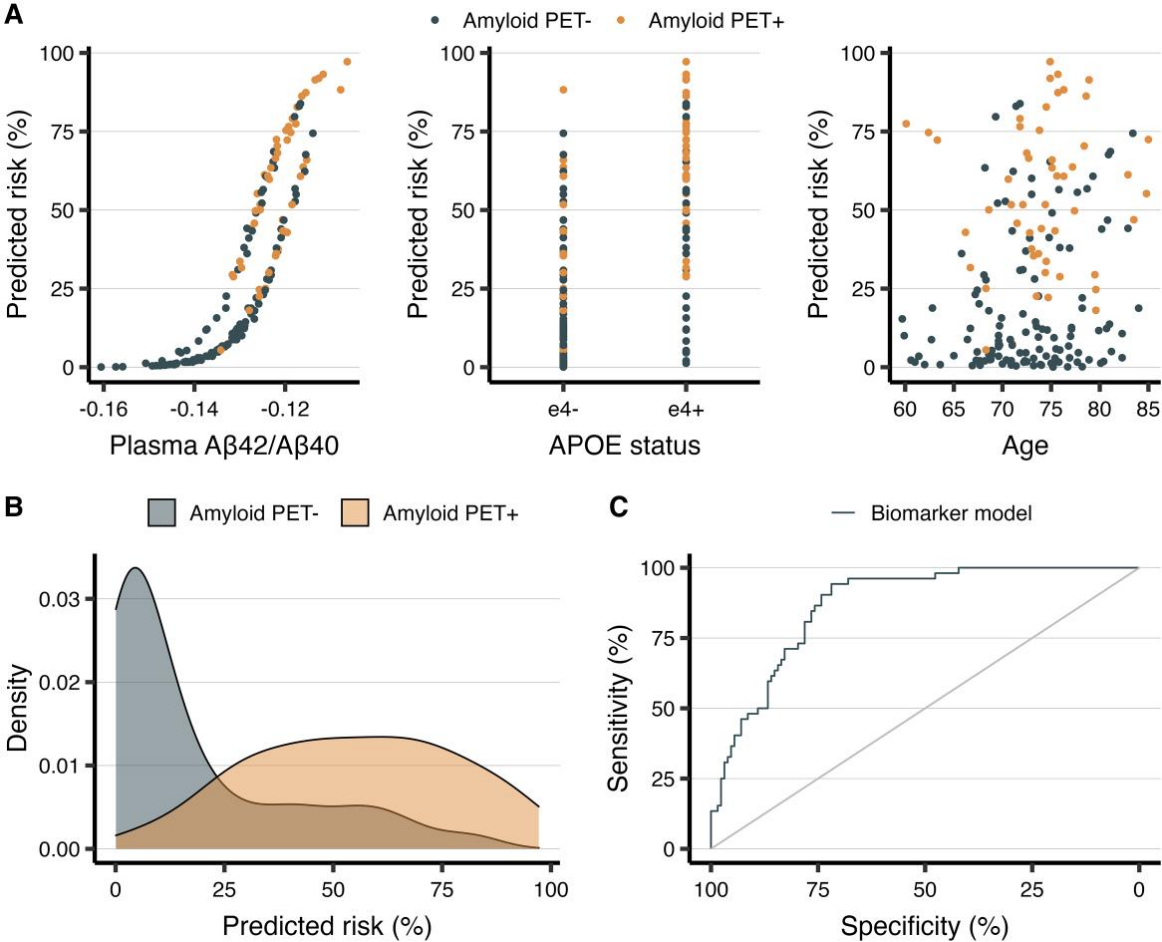
**Ability to estimate Aβ PET status from plasma biomarkers.** A logistic regression model was fit to predict Aβ PET status from age, plasma Aβ42/Aβ40 and *APOE* ε4 status. The effect of each variable in the model is shown for all individuals in subplot **A** (note that the upper line in the far-left subplot shows the effect of positive *APOE* status), the distribution of predicted amyloid PET risk (i.e. the kernel density estimate based on the probability density function) derived from the logistic regression model is visualized in subplot **B**, and a ROC curve showing results of the fitted model is visualized in subplot **C**. Note that plasma Aβ42/Aβ40 values have been negated to improve interpretability so that higher levels are associated with worsening risk for Aβ PET positivity.

The logistic regression model with plasma Aβ42/Aβ40, *APOE* status and age as predictors showed high performance in predicting Aβ PET status [area under the curve (AUC) = 0.87, 95% CI = (0.82, 0.92)] with bootstrap sampling. Moreover, evaluating model performance in a predictive context using 5-fold cross-validation resulted in an out-of-sample AUC of 0.86 [95% CI (0.85, 0.88)]. In this combined model, there was a significant effect for plasma Aβ42/Aβ40 [odds ratio (OR) = 5.98 (3.27, 12.32), *P* < 0.0001] and *APOE* status [OR = 3.67 (1.64, 8.46), *P* = 0.002], but not age [OR = 1.11 (0.73, 1.69), *P* = 0.62]. The effect of each variable on Aβ PET status is visualized in Fig. 2A, the distribution of predicted Aβ PET risk separated by true Aβ PET status is visualized in Fig. 2B, and a receiver operating characteristics (ROC) curve is shown in Fig. 2C.

Restricting the logistic regression analysis to CN participants led to only a slight drop in the performance of the combined model for predicting amyloid PET status [AUC = 0.84 (0.75, 0.92)], although the effect of plasma Aβ42/Aβ40 was still significant [OR = 4.21 (1.93, 11.02), *P* = 0.0011] and the effect of *APOE* status trended towards significant [OR = 1.55 (0.92, 2.64), *P* = 0.098].

### Relative risk approach to pre-screening

**Figure 3:**
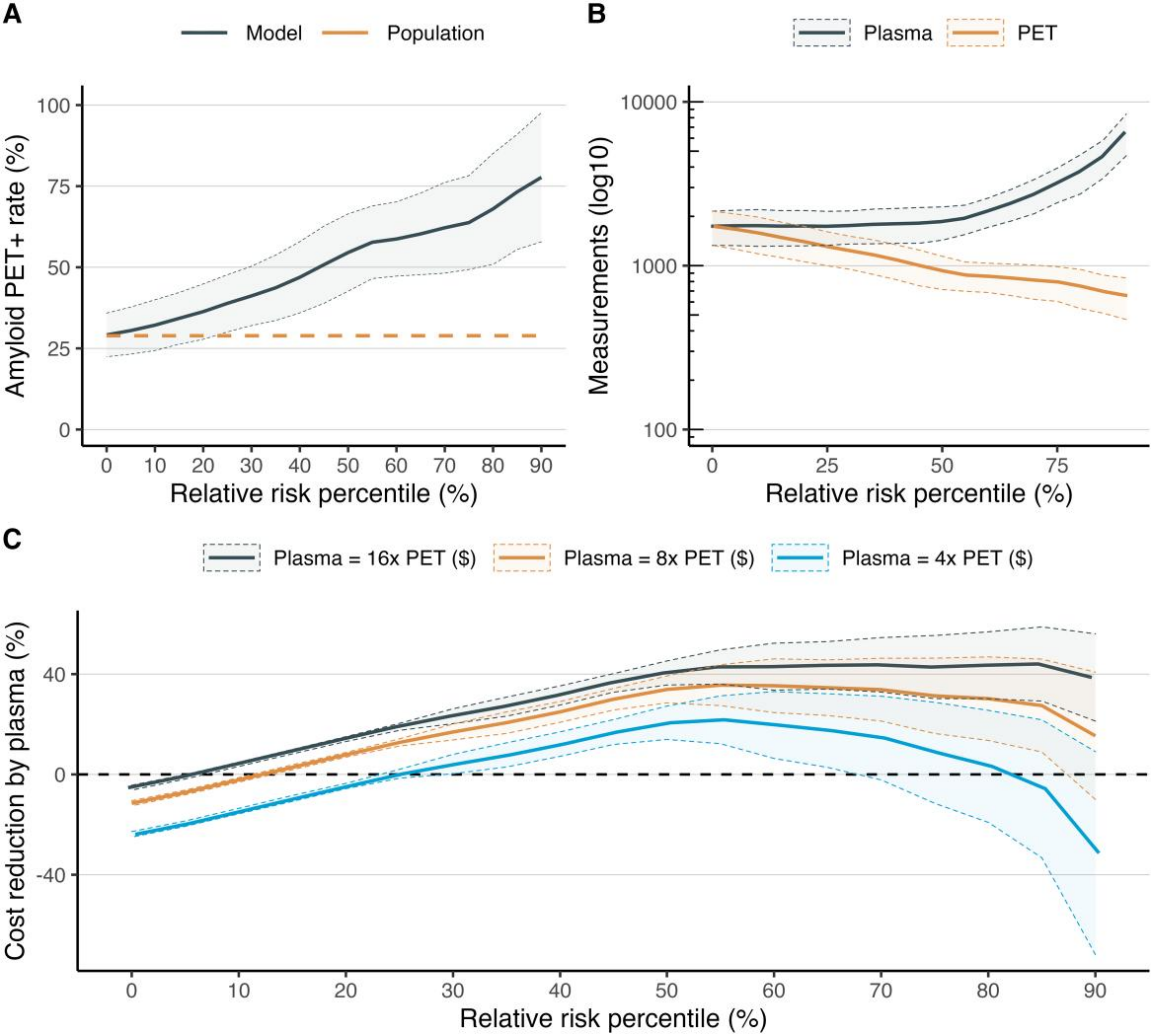
**Pre-screening with a relative risk threshold approach.** This figure shows the effect of different thresholds for relative risk (i.e. predicted probability from the logistic regression model normalized across the study population) on trial recruitment. Panel **A** shows the Aβ PET + rate as a function of relative risk (i.e. the *lower* threshold, so 0% means everyone gets a PET scan). Panel **B** shows the trade-off between the total number of tests (on log10 scale) needed in the pre-screening phase (i.e. plasma) versus the screening phase (i.e. PET) for a trial with 500 PET + CU participants. Panel **C** shows the total cost savings by pre-screening across different cost ratios. For example, a trial using the relative risk threshold of 25% would have an Aβ PET + rate of 38.6% and a threshold of 75% would have an Aβ PET + rate of 64.2%. The expected Aβ PET + rate with no pre-screening is 28.6%.

When using a relative risk approach to pre-screening where predicted Aβ PET risk was normalized based on percentiles derived from the entire population, the expected Aβ PET + rate increased from 28.9% with no pre-screening (i.e. the overall rate in the study population) to 38.7% [CI (29.8, 47.7); *P* < 0.0001 versus no pre-screening] with a 25th percentile risk cutoff (i.e. the 75% of individuals with highest estimated Aβ PET risk relative to others in the pre-screened population are invited for PET scanning), increased to 54.6% [CI (43.0, 66.2); *P* < 0.0001] with a 50th percentile risk cutoff, and increased to 63.7% [CI (48.8, 78.6); *P* < 0.0001] with a 75th percentile risk cutoff. These results are displayed in Fig. 3A.

In practical terms, recruiting 500 Aβ PET + CU individuals without plasma pre-screening would therefore require an estimated 1749 Aβ PET scans to fulfil recruitment. Meanwhile, using plasma biomarkers for pre-screening with a relative risk approach would require only 1312 PET scans (∼25% reduction) with a 25th percentile risk cutoff, 926 PET scans (∼47% reduction) with a 50th percentile risk cutoff and 796 PET scans (∼54% reduction) with a 75th percentile risk cutoff. However while no plasma measurements would be required without plasma pre-screening, 1750 plasma measurements would be required with a 25th percentile risk cutoff, 1850 plasma measurements with a 50th percentile risk cutoff and 3184 plasma measurements with a 75th percentile risk cutoff. These results are displayed in Fig. 3B.

When assuming that Aβ PET scanning would be four-times (4×) as expensive as plasma biomarker measurement, there were no significant cost savings by employing pre-screening with the plasma panel and a 25th percentile risk cutoff [Δcost = −8.7% (−12.1, 29.5), *P* = 0.18], or a 75th percentile risk cutoff [Δcost = −0.27% CI (−1.3, + 5.6), *P* = 0.05], but there was a significant cost saving with a 50th percentile risk cutoff [Δcost = −20.8% CI (−26.9, −14.7), *P* < 0.001]. However, plasma pre-screening always resulted in significant cost savings when PET scanning was assumed to be at least 8 × as expensive as plasma measurement [Δcost = −31.3% (−46.4, −16.2), *P* = 0.002 with a 25th percentile risk cutoff; Δcost = −33.9% (−38.9, −28.9), *P* < 0.001 with a 50th percentile risk cutoff; Δcost = −12.8% (−14.3, −11.2), *P* < 0.001 with a 75th percentile risk cutoff]. When Aβ PET scanning was assumed to be 16 × as expensive as plasma biomarker measurement, the savings were strongly significant for plasma [Δcost = −42.7% (−55.3, −30.2), *P* < 0.001 with a 25th percentile risk cutoff; Δcost = −40.6% (−45.3, −35.9), *P* < 0.001 with a 50th percentile risk cutoff; Δcost = −18.9% (−20.4, −17.5), *P* < 0.001 with a 75th percentile risk cutoff]. These results are displayed graphically in Fig. 3C.

### Absolute risk approach to pre-screening

**Figure 4:**
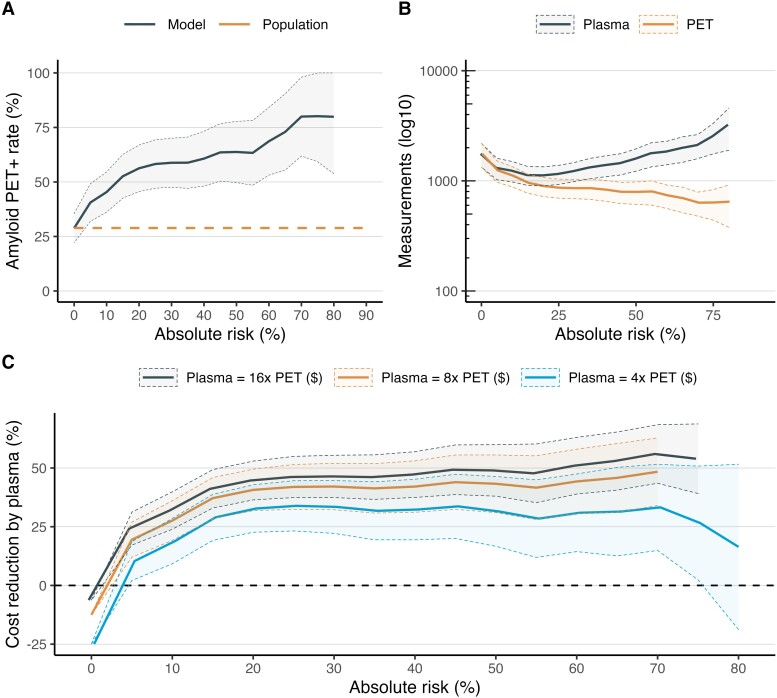
**Pre-screening with an absolute risk threshold approach.** This figure shows the effect of different thresholds for absolute risk (i.e. raw predicted probability from the logistic regression model) on trial recruitment. Panel **A** shows the Aβ PET + rate as a function of the absolute risk (i.e. the *lower* threshold, so a 0% risk cutoff means everyone gets invited for a PET scan). Panel **B** shows the trade-off between the total number of tests (on log10 scale) needed in the pre-screening phase (i.e. plasma) versus the screening phase (i.e. PET) for a trial with 500 PET + CU participants. Panel **C** shows the total cost savings by pre-screening across different cost ratios. For example, a trial with absolute risk threshold of 25% would have an Aβ PET + rate of 58.0% and a threshold of 75% would have an Aβ PET + rate of 80.3%. The expected Aβ PET + rate with no pre-screening is 28.6%.

With an absolute risk approach to pre-screening where an unnormalized predicted probability of Aβ PET positivity was used, the expected Aβ PET positivity rate increased from 28.9% with no plasma pre-screening (i.e. the overall rate in the study population) to 58.3% [CI (47.5, 69.2); *P* < 0.0001 versus no pre-screening] with a 25% risk cutoff (i.e. individuals with at least a 25% predicted probability of being Aβ PET + would be invited for PET scanning), increased to 63.8% [CI (50.5, 77.1); *P* < 0.0001] with a 50% risk cutoff, and increased to 80.2% [CI (58.9, 100); *P* < 0.0001] with a 75% risk cutoff. These results are displayed in Fig. 4A.

In practical terms, recruiting 500 Aβ PET + CU individuals without plasma pre-screening would require an estimated 1749 Aβ PET scans to fulfil recruitment as reported above. Meanwhile, using plasma biomarkers for pre-screening with an absolute risk approach would require only 865 PET scans with a 25% risk cutoff, 792 PET scans with a 50% risk cutoff and 637 PET scans with a 75% risk cutoff. And while no plasma measurements would be required without plasma pre-screening, 1153 plasma measurements would be required with a 25% risk cutoff, 1585 plasma measurements with a 50% risk cutoff, and 2548 plasma measurements with a 75% risk cutoff. These results are displayed in Fig. 4B.

Additionally, when assuming that Aβ PET scanning would be four-times (4×) as expensive as plasma biomarker measurement, there were significant cost savings by employing pre-screening with the plasma panel and a 25% risk cutoff [Δcost = −33.5% CI (−44.2, −22.4), *P* < 0.001 compared to no pre-screening], with a 50% risk cutoff [Δcost = −31.7% CI (−46.9, 16.5), *P* < 0.001] and with a 75% risk cutoff [Δcost = −26.6% CI (−50.9, −2.3), *P* = 0.028]. The same results were found when Aβ PET scanning was assumed to be 8 × and 16 × more expensive than plasma measurement. These results are displayed graphically in Fig. 4C.

### Sensitivity analysis of model predictors

The primary aim of our analysis was to expand on the use of a combination of plasma Aβ42/Aβ40, *APOE* status and age according to previously published work.^11^ However, as shown above, age was not a significant predictor in our fitted model, so we conducted a sensitivity analysis using plasma Aβ42/Aβ40 and *APOE* status as predictors, but without age.

For pre-screening, the results found using the relative risk approach were almost identical to those found when also including age (see Supplementary Fig. 1). In terms of performance of the logistic regression model, the model with only plasma Aβ42/Aβ40 and *APOE* status performed similarly to the full model [AUC = 0.868, 95% CI (0.817, 0.920)]. Fitting a logistic regression model with only *APOE* status and age led to a significantly worse model performance than the model with plasma Aβ42/Aβ40 [AUC = 0.734, 95% CI (0.654, 0.814)].

## Discussion

We demonstrated that a blood-based biomarker panel including plasma Aβ42/Aβ40, *APOE* status and age could be employed in the pre-screening phase of Alzheimer’s disease clinical trial recruitment to reduce future costs associated with Aβ PET screening. A sensitivity analysis also showed that plasma Aβ42/Aβ40 alone without age may even be equally as effective while removing plasma Aβ42/Aβ40 results in clearly worse performance. This suggests that the contribution of plasma biomarkers is clearly beneficial for pre-screening, while the role of age remains unsettled in the context of highly selected cohorts.

In the pre-screening phase, the ideal biomarkers are those which are non-invasive and inexpensive to collect, thereby allowing for measurement in a large number of individuals.^17^ For secondary prevention trials of Alzheimer’s disease, an important pre-requisite to recruitment is the identification of individuals who are at high risk of having amyloid pathology and are therefore likely to have an abnormal Aβ PET scan.

Our results demonstrated that accessible biomarkers can effectively identify high-risk and low-risk individuals and that pre-screening is cost-effective under the assumption that PET scanning is at least 4 × –8 × as expensive as plasma biomarker measurement. The expectation of PET scanning being 4 × as expensive as plasma biomarker measurement appears quite conservative given the high cost of Aβ PET scanning (considering both costs for equipment, consumables and staff) compared with the high competition for developing plasma biomarkers which may place downward price pressure.^18^ For example, an Aβ PET scan costs between $5000 and $8000 in clinical practice and can be higher in a research setting, while a blood test costs approximately $500–$1000.^18^ With the expectation of PET scanning being 16 × more expensive than plasma measurement, total costs of patient recruitment were estimated to be reduced by more than 40%. In reality, the PET-plasma cost ratio will likely vary depending on the situation and reducing the number of PET scans may also be beneficial in non-monetary ways considering the radiation and time consumption associated with PET scanning. To note, this cost-benefit analysis was done in terms of the relative cost between PET and plasma measurement to ensure generalizability of our results across clinics or locations where the absolute costs of such tests may differ greatly (e.g. between Europe and the US).

Our cost-benefit analysis suggests that the optimal pre-screening risk threshold is around 50% in terms of relative risk (i.e. individuals in the top 50% of predicted risk levels relative to those in a similar population) and 20% in terms of absolute risk (i.e. individuals with at least a 20% probability of being Aβ PET+). In the relative risk scenario, around half of the individuals who are pre-screened using plasma would be invited to receive an Aβ PET scan. This threshold increased the likelihood of Aβ PET positivity from 28.9 to 54.6%. Since almost every second scan would still be negative with this approach, these results suggest that simply maximizing positive predictive value or negative predictive value is not necessarily the optimal strategy for biomarker-based screening in a CU population. The size of the clinical trial population is also likely to play a role as larger fixed costs are more readily tolerated with larger trials, but a full exploration of this factor was outside the scope of the current analysis.

Previous efforts in the area of reducing unnecessary Aβ PET scans have considered widely available biomarker modalities such as cognition and MRI.^19–21^ Other studies have also investigated how Aβ risk assessment using simple cognitive tests and demographics can reduce the economic burden of PET scanning.^22,23^ However, our study contributes to the existing literature in its analysis of how a novel but readily available plasma biomarker reflecting amyloid pathology can be leveraged together with *APOE* status and Aβ PET scanning during trial recruitment to reduce overall costs of trial inclusion.^24,25^ Our results are also novel in the targeting of CU individuals at the earliest stages of Alzheimer’s disease pathological development, where recruitment is likely to be difficult without effective pre-screening tools. Our study built upon a previously suggested combination of plasma Aβ42/Aβ40, *APOE* status and age, but in a sensitivity analysis, we noted that the cost-benefit results were largely unchanged using a more sparse model with only plasma Aβ42/Aβ40 and *APOE* status. This may suggest that age is not a necessary component in prediction models in CU populations when efficient biochemical tests are employed. Alternatively, the lack of an effect of age in our study (in contrast to Ref. ^11^) could be a cohort-specific effect. Besides a more thorough evaluation of model variables, additional novel contributions of our study include an emphasize on cost-benefit calculations across a variety of inclusion thresholds and relevant clinical trial scenarios.

One important previous study did look at a similar panel of plasma Aβ42/Aβ40 and *APOE* by evaluating its ability to predict and screen for Aβ PET status.^26^ The result presented in that article was validated nicely by our model since the AUC values were similar between studies (0.84 for their study, 0.87 for our study). The main addition of our study was the use of a more sensitive and well-validated MS-based assay for measuring plasma Aβ42/Aβ40 that is already being implemented for clinical care and clinical trial recruitment.

Practically speaking, we explored two contrasting methods of applying biomarker-based algorithms in a pre-screening context. The first method is to use the *relative risk* for screened individuals, where the predicted risk for amyloid PET positivity for each individual is compared to all other screened individuals within a batch of biomarker samples. After biomarkers have been analysed, only a certain percentage of individuals in terms of predicted risk for amyloid positivity are carried forward to PET. The advantage of this approach is that it provides a consistent sample size for downstream screening and is likely to be more robust against the variability of assays or demographic compositions between sites. Ensuring equitable inclusion in clinical trials is a major goal given the acknowledgement that biomarker cutoffs are not necessarily applicable across different socio-economic groups.^27,28^ When using the relative risk approach, however, there may be a delay between plasma analysis and the determination of eligibility for PET scanning for the individual participants.

The second option would be to use the absolute risk of Aβ PET positivity for each screened individual. Here, the statistical model is used to generate a predicted Aβ PET risk score based on each individual’s biomarker data, using previously derived model specifications. Only e.g. those with >75% absolute risk of Aβ positivity are carried forward to PET. This approach would require highly standardized assays and generalizable clinical prediction models. The advantage of this approach is that it allows for greater control of positive predictive value or negative predictive value for inclusion of actually amyloid PET + participants compared to the relative risk strategy. This approach also allows for quicker differentiation of participants into those who should move on to PET scanning versus those who are not eligible but requires careful calibration and monitoring of prediction models.

Regardless of whether a relative or absolute risk approach is employed, the use of an MS-based plasma assay means that plasma samples would have to be shipped to one (or at most a few) central locations which have the expertise to measure such samples. This is a clear drawback compared to automated platforms or immunoassays where samples can be measured on site. However, to date, there does not appear to be any non-MS-based plasma Aβ assays which meet suitable diagnostic standards for Aβ PET screening.^14^

The strengths of our study include a well-characterized cohort with longitudinal follow-up over highly relevant timeframes for secondary prevention trials of Alzheimer’s disease.^29^ Our study also features the latest plasma Aβ42/Aβ40 assay which has been shown to be effective in estimating Alzheimer’s disease-related outcomes on its own and combined.^30–36^ This assay, along with *APOE* status and age, combines to make up a well-validated test which has been certified for use in predicting risk for amyloid pathology. The fact that this panel is already commercially available for clinical use and is implemented for clinical trial screening makes it important to validate in independent cohorts. Future studies may further compare this panel with models that also incorporate other promising Alzheimer’s disease biomarkers under development.

In terms of generalizability, the model was evaluated using both bootstrap sampling and cross-validation, which showed good concordance between performance on train and test samples. This indicates that the model is likely to generalize to new data, although performance would depend on assay variability and reliability.

The limitations of our analysis are a relatively small cohort in relation to the heterogeneity likely to be found in a CU elderly population, even if the size of our cohort relative to previous studies is quite large. A larger collection of CU individuals pulled from cohorts unrelated to Alzheimer’s disease could provide important information towards this end in the future.

Additionally, this study included participants who were recruited specifically as part of an Alzheimer’s disease-related study, meaning that the performance of our model may be optimistic compared to a true population screening scenario where specific expertise in plasma biomarkers may not be available. However, when we excluded SCD participants from the analysis to simulate a true population-based screening scenario, the performance of the model dropped only slightly. This indicates that our model was not overly influenced by the inclusion of SCD participants.

We restricted the analyses to a readily available plasma biomarker panel, but the addition of other biomarkers may also be relevant. The optimal biomarker model may likely depend on both the target population and outcome of interest, as previous research has shown that the utility of specific plasma biomarkers varies greatly depending on disease stage.^37^ Cerebrospinal fluid biomarkers could also theoretically be used to screen for Aβ PET status, as they show much stronger association than plasma biomarkers. However, they are not as accessible or inexpensive as plasma biomarkers, making their utility for Aβ PET screening limited.

## Supplementary Material

fcad015_Supplementary_Data

## Data Availability

Anonymized data will be shared by request from a qualified academic investigator for the sole purpose of replicating procedures and results presented in the article if data transfer is in agreement with EU legislation on the general data protection regulation and decisions by the Ethical Review Board of Sweden and Region Skåne, which should be regulated in a material transfer agreement. Code in the R programming language to reproduce the logistic regression modelling and the screening analysis is available in Supplementary Text 1.

## Abbreviations

Aβ =: Beta-amyloid protein
APOE =: apolipoprotein E
AUC =: area under the curve
BioFINDER =: Biomarkers for Identifying Neurodegenerative Disorders Early and Reliably
CI =: confidence interval
CN =: cognitively normal
CU =: cognitively unimpaired
MS =: mass spectrometry
OR =: odds ratio
SCD =: subjective cognitive impairment
SUVR =: standardized uptake value ratio
