# Supplementary material for: Plasma amyloid-β42/40 and apolipoprotein E for amyloid PET pre-screening in secondary prevention trials of Alzheimer’s disease
Source: Brain Commun. 2023 Jan 24;5(2):fcad015. doi: 10.1093/braincomms/fcad015 (PMC10012324; doi:10.1093/braincomms/fcad015)
Supplement: fcad015_Supplementary_Data [file fcad015_supplementary_data.pdf]

Supplementary Material

Plasma Aβ42/Aβ40 and *APOE* for amyloid PET pre-screening in secondary prevention trials of Alzheimer's disease

Nicholas C. Cullen[1], Shorena Janelidze[1], Erik Stomrud[1,2], Randall J. Bateman[3], Sebastian Palmqvist[1,2], Oskar Hansson[1,2,*], Niklas Mattsson-Carlgren[1,4,5.*]

[1]Clinical Memory Research Unit, Department of Clinical Sciences Malmö, Faculty of Medicine, Lund University, Lund, Sweden

[2]Memory Clinic, Skåne University Hospital, Malmö, Sweden

[3]Department of Neurology, Washington University School of Medicine, St. Louis, Missouri, U.S.A.

[4]Department of Neurology, Skåne University Hospital, Lund, Sweden

[5]Wallenberg Center for Molecular Medicine, Lund University, Lund, Sweden

* Oskar Hansson and Niklas Mattsson-Carlgren contributed equally to this work.

Supplementary Figure 1 – Pre-screening with plasma Aβ42/Aβ40 and *APOE* status only

This figure shows the effect of different thresholds for relative risk (i.e., predicted probability from a logistic regression model normalized across the study population) on trial recruitment. Here, the logistic regression model to predict amyloid PET status included plasma Aβ42/Aβ40 and *APOE* status only, not age. Panel A shows the amyloid PET+ rate. Panel B shows the trade-off between the total number of tests (on log10 scale) needed in the pre-screening phase (i.e., plasma) versus the screening phase (i.e., PET) for a trial with 500 PET+ CU participants. Panel C shows the total cost savings by pre-screening across different cost ratios.

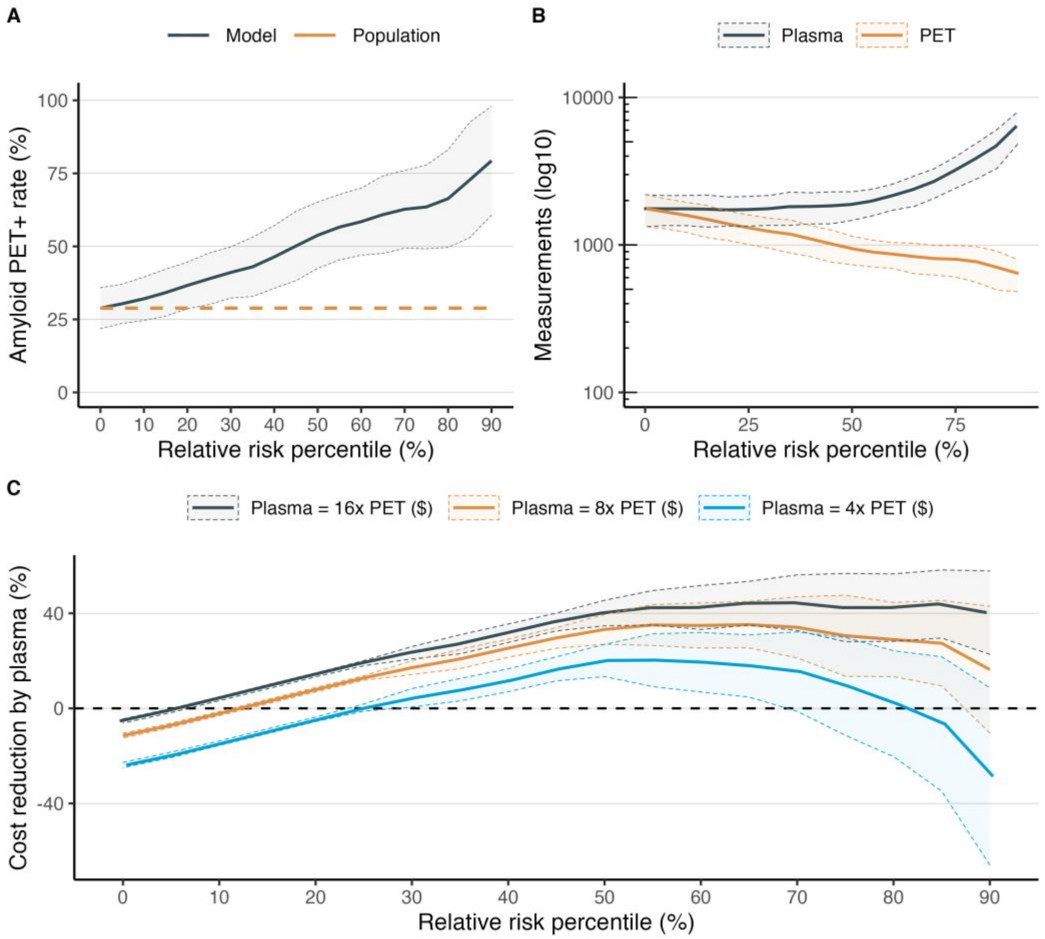

Supplementary Text 1 – Example analysis code in R

This supplementary text contains example code to reproduce the logistic regression modelling and the screening analysis using the automated biomarker analysis ("aba") package in the R programming language.

```
model <- df %>% aba_model() %>%
  set_groups(everyone()) %>%
  set_outcomes(AB.PET.STATUS,
             .labels = c('Amyloid PET status')) %>%
  set_predictors(
    c(PLASMA_ABETA_bl, APOE, AGE_bl),
    .labels = c('Plasma Aβ42/Aβ40 + APOE + Age')
  ) %>%
  set_stats(
    stat_glm(std.beta=T)
  ) %>%
  fit()

model_summary <- model %>% aba_summary()

model_screen <- model %>%
  aba_screen(
    threshold = seq(0.0, 0.9, by = 0.05),
    cost_multiplier = c(4, 8, 16),
    include_n = 500,
    ntrials = 10,
    verbose = T,
    risk_type = 'relative'
  )
```
